# Comparison of robotic and open radical prostatectomy: Initial experience of a single surgeon

**DOI:** 10.12669/pjms.37.1.2719

**Published:** 2021

**Authors:** Adnan Simsir, Fuat Kizilay, Bayram Aliyev, Serdar Kalemci

**Affiliations:** 1 Dr. Adnan Simsir, Department of Urology, Ege University School of Medicine, Izmir, Turkey; 2 Dr. Fuat Kizilay, Department of Urology, Ege University School of Medicine, Izmir, Turkey; 3 Dr. Bayram Aliyev, Department of Urology, Ege University School of Medicine, Izmir, Turkey; 4 Dr. Serdar Kalemci, Department of Urology, Ege University School of Medicine, Izmir, Turkey

**Keywords:** Robot-assisted radical prostatectomy, Retropubic radical prostatectomy, Prostate cancer, Oncological results, Functional results

## Abstract

**Objective:**

In this study, we aimed to make a comprehensive comparison of the first hundred robot-assisted radical prostatectomy (RARP) and open retropubic radical prostatectomy (RRP) cases of a single surgeon in a high-volume center.

**Methods:**

Preoperative, perioperative and postoperative data were collected retrospectively. Perioperative, oncological data and functional results in the first year were compared between the two groups. There were 204 RARPs between January 1, 2014 and December 31, 2019, and 755 RRPs between April 1, 2007 and December 31, 2019.

**Results:**

While the operation time was in favor of the open group (117 vs 188 min, p<0.001), the estimated blood loss (328 vs 150 ml, p<0.001), blood transfusion rate (12 vs 2, p=0.021), and re-operation rate (6 vs 0, p=0.001) were in favor of the robotic group. Mean length of hospital stay (5.4 vs 3.1, p<0.001), urine leak rate (11 vs 2, p=0.033), complication rate (37 vs 16, p=0.018), and the 12th month continence rate (67 vs 85, p=0.002) were better in the robotic group.

**Conclusions:**

RARP may provide better perioperative outcomes and lower complication rates after the surgeon factor is eliminated in the early period. Since our case group includes the initial 100 patients, studies with larger patient groups with longer follow-up are needed to adapt these early results to general outcomes.

## INTRODUCTION

Radical prostatectomy is the main recommended surgical treatment for clinical localized prostate cancer, which provides long-term oncological control. Open retropubic radical prostatectomy (RRP) is a conventional surgical method that provides excellent success rates. However, the complexity of the pelvic anatomy, the prostate being a deep and hard-to-reach organ forced the surgeons to develop novel techniques.^1^ For this purpose, minimally invasive methods have been developed and laparoscopic surgery has been initiated. However, the steep learning curve and difficult technique of laparoscopic prostatectomy greatly prevented the spread and frequent use of this technique. To overcome these difficulties, robot-assisted radical prostatectomy (RARP) technique has been used since the early 2000s. It was first used in Germany in 2001 then refined by Menon et al in United States.^2^,^3^ Due to the enlarged view and advanced articulation arms of robots, it is predicted that it provides more careful prostate resection and better functional results by preserving the neurovascular bundle.

Although up to 85% of radical prostatectomies in the United States are performed using robotic technique, the rate of RRP in the world is still considerably high due to the cost and availability of the robots.^4^ There are many studies that compare the two techniques extensively in terms of surgery, oncology and function, as well as specific studies that compare only in terms of the positive surgical margin or anesthesia method.^5^-^7^ Publications on the comparison of open and robotic technique are more limited in number and include more health resources and cost analysis.^8^,^9^ There are limited number of publications showing that the oncological and functional results of the two techniques are similar.^10^,^11^

While designing this study, we hypothesized that comparing the first 100 cases of a single surgeon would provide more consistent information in terms of reflecting the early results of the two techniques by eliminating the surgeon factor, and for this purpose we aimed to compare a comprehensive pre-, peri- and postoperative results of the two techniques.

## METHODS

**Fig.1:**
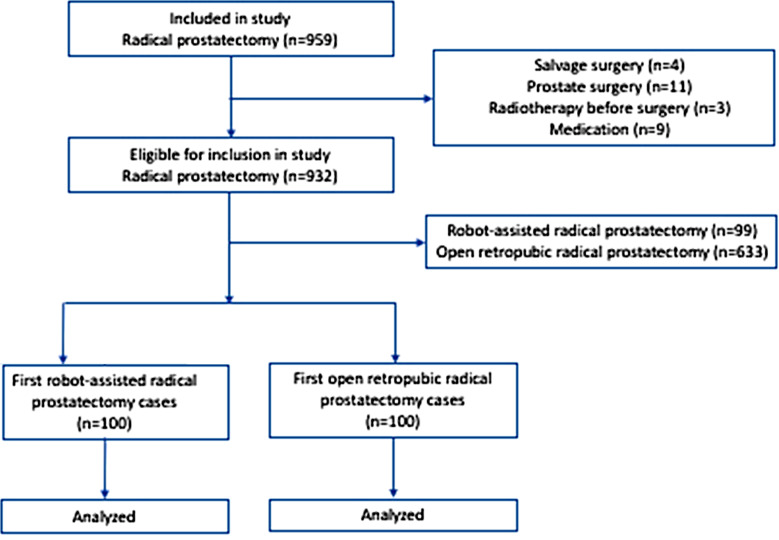
Flowchart for the study population.

Radical prostatectomy cases performed by a single surgeon from April 2007 to December 2019 were included in the study. The total case numbers of this surgeon were as follows according to robotic and open techniques and date ranges; there were 204 RARPs between January 1, 2014 and December 31, 2019, and 755 RRPs between April 1, 2007 and December 31, 2019. Institutional Review Board approval number and date: 2020/012.38-10.03.2020. Our study, which has a retrospective case-control structure, covered cases in a tertiary university hospital over a 12-year period. Patients who had undergone salvage surgery, had undergone a surgical procedure for prostate, received radiotherapy before surgery and used a medication affecting lower urinary tract symptoms were excluded. The flow chart of the study is summarized in Fig.1.

Perioperative and oncological results between RRP and RARP were compared. Blood transfusion rate, mean length of hospital stay, complication rate, urine leakage rate, the need for re-operation, and mean blood loss were compared between the two groups. In addition, the positive margin status, which is an important factor in predicting cancer-free survival, mean operative time, and the erectile function status and incontinence rate at 12 months were also compared.

### Operation Techniques:

### Robot-assisted radical prostatectomy

All robotic surgeries were performed with da Vinci^®^ Robotic Surgical Systems (Intuitive Surgical). Surgical interventions with the robotic technique were performed with the posterior approach described by Guillonneau and Vallancien in the Montsouris technique in 2000.^12^ Under intratracheal general anesthesia, the patient was placed in the maximum trendelenburg position and an 18 Fr urethral catheter was introduced. Skin and subcutaneous incision was made at the level of 2 cm superior to the umbilicus. Veress^®^ needle was entered into the abdominal cavity from this area, CO_2_ insufflation was started. 12 Fr camera port, other robot ports and assistant port were entered. Following this, docking was done. In the posterior approach, the vas deferens and seminal vesicles are dissected before the Retzius space is created. A U-shaped incision was made in the peritoneum 1-1.5 cm above the rectum over the vas deferens. The areolar tissue in the region was dissected in order to locate and dissect the vas deferens. The seminal vesicles posterior to the vas deferens were also located and separated from the surrounding tissues by blunt and sharp dissections. The fascial sheath around the prostate was dissected. The lateral pelvic fascia was sharply incised along the anterolateral prostate. It was temporarily occluded using Weck clips and sutured after removal of the prostate. The ipsilateral seminal vesicle was grasped with fourth arm and suspended to clearly expose the pedicle. After cutting the pedicle, the posterolateral connections between the neurovascular bundle and the prostate were sharply incised with scissors. The endopelvic fascia is incised and opened. Finally, the resulting levator ani fibers were removed. Right and left puboprostatic ligaments were cut. The dorsal venous complex was passed with 40 mm 0 Vicryl^®^ stitches and knotted. Then, an incision was made with the monopolar scissors to separate the bladder and the prostate floor. Then, the incision was made through the bladder-prostate border and the bladder neck was cut. The catheter was removed and the posterior urethra was cut. The surgical specimen was then removed and placed into a laparoscopy bag or left in the pelvis. A secure, mucosa-to-mucosa, vesicorurethral anastomosis was created using a continuous suture. After the anastomosis was created, a 22 Fr Foley catheter was introduced and the bladder was filled to check for anastomotic leakage.

### Retropubic radical prostatectomy

All RRP cases were performed in accordance with the technique Reiner and Walsh developed primarily to control the dorsal venous complex and protect the neurovascular bundle in 1979.^13^ Under intratracheal general anesthesia, an 18 Fr Foley catheter was inserted and the skin, subcutaneous tissue, and muscles were passed through the suprapubic incision. Then the endopelvic fascia was opened, the venous plexus was ligated with 0 Vicryl^®^ and cut. The urethra was released, suspended and cut. The prostate was released from Denonvilier fascia with sharp and blunt dissections. Bilateral ductus deferens were clamped and cut, the right seminal vesicle, left seminal vesicle, along with the prostate, were removed, and the bladder neck was narrowed with a 2/0 Vicryl Rapid^®^ suture. A 22 Fr Foley catheter was introduced. Urethrovesical anostomosis was cretaed in six quadrants with 2/0 Monocryl^®^ suture. One drain was placed on the operation site, fasia was closed with 0 PDS^®^, subcutaneous tissue was closed with 2/0 Vicryl Rapid^®^, and the skin was closed with 2/0 silk suture.

### Endpoints and measurement criteria

Comparison of perioperative and oncological results between the two groups was the primary endpoint of the study, and the comparison of functional results was the secondary endpoint. The number of erections reported by the patient, sufficient for penetration with or without erectogenic drugs, as well as the number of urine pads used daily by patients were recorded. Clavien system was used for classification of complications: (I) slight deviation from the norm not requiring treatment, (II) slight deviation from the norm requiring pharmacological treatment, (IIIa) invasive intervention without general anesthesia, (IIIb) invasive intervention under general anesthesia.^14^ Urinary leakage was defined as urine leakage from the urethrovesical anastomosis, which was defined by the clinical or radiological evidence of urine leakage in undrained patients and five times the serum level of fluid creatinine drainage in drained patients. In addition, biochemical recurrence status was evaluated according to the PSA level in the first year. A PSA value above 0.2 ng/mL was considered as biochemical recurrence.

### Statistical Analysis

Continuous variables were presented as mean and standard deviation, categorical variables as percentage. Numerical parameters between the two groups were compared with Student’s t-test or the Mann–Whitney U test. Chi-squared test was used to compare nominal data. A p <0.05 value was accepted for statistical significance.

## RESULTS

The data of first 100 RARP and RRP cases that met the inclusion criteria were analyzed. Demographic data, PSA values, biopsy Gleason scores and preoperative erectile functions of the two groups were similar (p>0.05). There was no significant difference between the patients’ digital rectal examination findings, pathological Gleason scores and preoperative hemoglobin values (p>0.05). The comparison of the demographic characteristics and preoperative data of the two groups is summarized in Table-I.

**Table-I:** Demographic data of patients.

Variables	Open (n=100)	Robotic (n=100)	P value
Age	62.8 (48-76)	64.6 (47-79)	0.519
**DRE**			
Non-palpable	71 (71%)	73 (73%)	0.480
Palpable	29 (29%)	27 (27%)	
PSA value at the time of diagnosis (ng/mL)	7.82 (5.1-17.1)	7.20 (4.8-16.9)	0.683
**Biopsy Gleason score**			
3+3	18 (18%)	22 (22%)	
3+4	36 (36%)	38 (38%)	0.112
4+3	28 (28%)	26 (26%)	
8	12 (12%)	10 (10%)	
9-10	6 (6%)	4 (4%)	
**Pathological Gleason score**			
3+3	15 (15%)	17 (17%)	
3+4	40 (40%)	42 (42%)	0.061
4+3	31 (31%)	30 (30%)	
8	11 (11%)	8 (8%)	
9-10	3 (3%)	3 (3%)	
Preoperative hemoglobin value	12.9 (11.2-14.9)	13.2 (11.8-14.2)	0.721
**Preoperative erectile function**			
Sufficient for intercourse	83 (83%)	78 (78%)	0.089
Not sufficient for intercourse	17 (17%)	22 (22%)	

DRE:digital rectal examination; PSA:prostate-specific antigen

While the operation time was in favor of the open group (117 vs 188 min, p<0.001), the estimated blood loss (328 vs 150 ml, p<0.001), blood transfusion rate (12 vs 2, p=0.021), and re-operation rate (6 vs 0, p=0.001) were in favor of the robotic group. While the operation time was in favor of the open group, all other perioperative data were in favor of the robotic group. Comparison of perioperative data is shown in Table-II.

**Table-II:** Comparison of Perioperative Results.

Variables	Open (n=100)	Robotic (n=100)	P value
Operation time (minutes)	117 (94-251)	188 (130-319)	<0.001
Estimated blood loss (mL)	328 (41-3280)	150 (38-1810)	<0.001
Blood transfusion	12 (12%)	2 (2%)	0.021
The need for re-operation	6 (6%)	0 (-)	0.001

While mean length of hospital stay (5.4 vs 3.1, p<0.001), urine leak rate (11 vs 2, p=0.033), complication rate (37 vs 16, p=0.018), and the 12th month continence rate (67 vs 85, p=0.002) were better in the robotic group, no significant difference was observed between the two groups in terms of surgical margin status (71 vs 79, p=0.590), bladder neck contracture (3 vs 5, p=0.810) and erectile function (52 vs 62, p=0.214) in the first year. Another factor predicting oncological results, PSA failure rates in the first year were similar between the two groups (7 vs 5, p=0.121). All postoperative results except for bladder neck contracture, surgical margin positivitity, and erectile function at 12 months were in favor of the robotic group. In terms of these data, there was no significant difference between the two groups. Comparison of postoperative data and functional results of the patients in the first year are shown in Table-III.

**Table-III:** Comparison of Postoperative and Functional Results.

Variables	Open (n=100)	Robotic (n=100)	P value
The length of hospital stay (days)	5.4 (3-12)	3.1 (2-5)	<0.001
Clavien complication	37 (37%)	16 (16%)	
Clavien I	13 (13%)	5 (5%)	
Clavien II	11 (11%)	8 (8%)	0.018
Clavien IIIa	10 (10%)	3 (3%)
Clavien IIIb	3 (3%)	-	
Urine leakage	11 (11%)	2 (2%)	0.033
*Surgical margin*			
Negative	71 (71%)	79 (79%)	0.590
Positive	29 (29%)	21 (21%)	
Biochemical recurrence	7 (7%)	5 (5%)	0.121
Bladder neck contracture	3 (3%)	5 (5%)	0.810
*Daily pad use at the 12th month*			
0	67 (67%)	85 (85%)	0.002
>1	33 (33%)	15 (15%)	
*Erectile function at the 12th month*			
Sufficient for intercourse	52 (52%)	62 (62%)	0.214
Not sufficient for intercourse	48 (48%)	38 (38%)	

## DISCUSSION

RRP, which is performed with a small incision, is the conventional surgical treatment of prostate cancer with tolerable postoperative pain and hospitalization time. Due to its marketing strategy and convenience to the surgeons, RARP has begun to be applied with increasing frequency, but a significant number of patients are still treated with RRP in many countries due to cost constraints.^15^ According to some authors, it seems no longer necessary to compare open and robotic prostatectomy, but this discussion will continue, given the reality that RRP is still done frequently.^16^ Radical prostatectomy, which is the standard treatment for men with a life expectancy of at least 10 years, may lead to significant complications such as bleeding, pain, incontinence, anastomosis stricture and erectile dysfunction, despite advances in the technique.

Thanks to the technical advantages provided by RARP, it is a question of whether it will reduce the complication rate compared to open surgery. However, there are conflicting results in the literature. In a prospective, comparative study, there was no significant difference in re-hospitalization and Clavien-Dindo 3b and higher complication rates, whereas in a multi-center, high-case study, the complication rate below Clavien-Dindo three was higher in the RRP group.^17^,^18^ In high-volume centers, complication rates are low with both techniques and are often in minor Clavien degrees. In a single-center retrospective analysis of approximately 14.000 cases, RARP has been shown to have slightly better complication rates than RRP.^19^ In our study, the complication rate in the RRP group was significantly higher. However, most of those were Clavien I-II class complications and no difference may be observed after completing the learning curve of the RRP. RRP and RARP were compared in a veteran’s affairs hospital and the study revealed that RARP provided better outcomes especially in perioperative parameters, but there was no significant difference in positive surgical margin rates and functional results.^20^ In this study, 153 RARPs were included in total, and it was stated that similar operation durations were provided after the first 100 RARPs. Docking and preparation stages in RARP may cause long operation time, especially during the learning curve period. It can be predicted that this period may also be shortened after the general accepted 40-cases learning curve. Similar operation times can be expected in subsequent cases since our study includes the first 100 cases.

Transfusion rates ranging from 8-30% in RRP have been reported in the literature.^21^,^22^ In another study involving both high and low volume centers, this rate was 16%.^17^ Undoubtedly, personal preferences, accepted transfusion thresholds and cardiac reserves of patients are important factors that determine these rates. In our study, the estimated blood loss in the RARP group and proportionally, the transfusion rate was lower. Perioperative bleeding may disrupt the appearance of the surgical site and prevent careful dissection and optimal performance of urethrovesical anastomosis. As a result, a higher rate of positive surgical margins and urine leakage may be expected. While the surgical margin rate was similar in our study, the rate of urine leakage was higher in the RRP group as a result of hemorrhage. In our study, PSA failure rates in the first year, another factor predicting oncological results, were similar between the two groups. These results reveal that the two methods have similar oncological safety in the early period.

It has been shown that the amount of blood lost during prostatectomy may cause acute kidney injury. It has been reported that blood loss and blood transfusion in RRP may increase renal ischemia and cause a higher rate of injury than RARP.^23^ However, in a more recent study, the incidence of acute renal injury was similar between the two groups, and it was underlined that this issue should be clarified through long-term prospective-randomized trials.^24^ Our study did not include the postoperative renal function data of patients, but none of them had any evidence of chronic renal failure in the postoperative period.

In radical prostatectomy, besides oncological results, achieving optimal functional results affecting the quality of life of patients are also very important. In a study analyzing the experience of a single surgeon in two techniques, no difference was found in terms of anastomosis leakage, 12-month continence rate, and bladder neck contracture.^25^ In our study, although the rate of urine leakage and continence rate was in favor of RARP, the rate of contracture was similar in the two groups. The previous study reflected the experience of a single surgeon over four years, and therefore, including cases that exceeded the learning curve may have resulted in similar results in both groups. In addition, the 6 foci urethrovesical anostomosis we performed in RRP (10, 12, 2, 4, 6, 8 o’clock alignment) allow a mucosa-to-mucosa anostomosis similar to that of RARP by allowing less foreign tissue to enter between the anostomosis line. On the other hand, we can suggest that the longer urethral length that can be achieved in RARP also leads to better continence rates.

According to the current Cochrane analysis, RARP appears to improve the quality of urinary and sexual life according to laparoscopic and open methods. RARP can reduce general surgical and major postoperative complications. RARP can provide less postoperative pain and shorter hospitalization time. In addition, the rate of blood transfusion may decrease with minimally invasive methods.^26^ Our findings, other than sexual results, coincide with the results of the Cochrane analysis. However, instead of a comprehensive inquiry form such as IIEF to evaluate sexual functions in the first year, our evaluation of erectile function as adequate or inadequate may explain the similar results in the two groups.

### Limitations of the study

It has a retrospective structure. Although the only surgeon experience removes the surgeon factor from the equation, the surgeon may have different abilities in open and robotic techniques. Also, adapting the experience of a single surgeon to all institutes may not be very realistic. The generally accepted recommended number of cases for learning curves of RRP and RARP are 250-1000 and 40, respectively.^27^ Although the number of cases in our study was not enough to complete the learning curve of the RRP, it should not be overlooked by the surgeons’ personal abilities. In addition to the cases performed at the same time, there were also cases done in different time frames. This may seem like a limitation for objective comparison of groups, but the longer learning curve compared to RARP despite early initiation of RRP may mask this difference. Another limitation is that we evaluated erectile and continence functions with short patient responses instead of validated questionnaire forms.

## CONCLUSIONS

We found that RARP may provide better perioperative and lower complication rates after the surgeon factor is eliminated in the early period. We also revealed that continence rate, which is an important functional outcome, is also better in the robotic group. Although RARP is growing in popularity with the push of the industry, it seems that RRP will continue to be performed for a long time, especially in countries with low socioeconomic level due to its high cost and difficulty in performing laparoscopic prostatectomy. Since our case group includes the initial 100 patients, studies with larger patient groups with longer follow-up are needed to adapt these early results to general outcomes.

### Author’s Contribution:

**AS, FK:** Conceived, designed and did statistical analysis & editing of manuscript

**AS, FK, BA & SK:** Did data collection and manuscript writing

**AS, FK & SK:** Did review and final approval of manuscript

**AS, FK, BA & SK:** Responsible and accountable for the accuracy of the study.
